# HPLC–DAD Analysis, Antimicrobial and Antioxidant Properties of Aromatic Herb *Melissa officinalis* L., Aerial Parts Extracts

**DOI:** 10.1007/s12161-022-02385-1

**Published:** 2022-08-23

**Authors:** Fahima Abdellatif, Samir Begaa, Mohammed Messaoudi, Adel Benarfa, Hamza Ouakouak, Aicha Hassani, Barbara Sawicka, Jesus Simal Gandara

**Affiliations:** 1 Laboratoire de Recherche Sur Les Produits Bioactifs Et La Valorisation de La Biomasse, Ecole Normale Supérieure Kouba, Algérie, B.P. 92, AlgerKouba Alger, Algeria; 2 Nuclear Research Centre of Birine, Djelfa, P.O. Box 180, 17200 Ain Oussera, Algeria; 3 University of Hamma Lakhdar El-Oued, P.O. Box, 789, 39000 El-oued, Algeria; 4 Center de Recherche Scientifique Et Technique en Analyses Physico-Chimiques (CRAPC)-PTAPC Laghouat, Laghouat, Algeria; 5 Department of Plant Production Technology and Commodities Science, University of Life Science in Lublin, Akademicka 15 str, 20-950 Lublin, Poland; 6 Nutrition and Bromatology Group, Department of Analytical Chemistry and Food Science, Faculty of Food Science and Technology, University of Vigo‐Ourense Campus, E32004 Ourense, Spain

**Keywords:** *Melissa officinalis*, Flavonoids, HPLC/DAD, Solvent extraction, Antioxidant activity, Antimicrobial activity, DPPH test

## Abstract

In order to enhance natural products value, *Melissa officinalis* (lemon balm) aerial part (leaves) has been studied in this work. Hence, the objective of this study is to determine the chemical composition of the studied plant polyphenols extracts using HPLC/DAD, as well as evaluate their flavonoid extracts’ antioxidant and antimicrobial activities using DPPH• and disk diffusion methods, respectively. The results of phenols chemical composition showed the existence of two phenolic acids, five flavonic aglycones and six heterosides, while the biologic results of the plant flavonoid extracts exhibited the existence of a good antioxidant and antimicrobial activities.

## Introduction

The botanical name of lemon balm is *Melissa officinalis* L., in pharmacopeia *Melissae foluim*; it is a plant that belongs to the labiate family. It is a fully aphyllous perennial plant, often very branched and characterized by their long stems (30 to 80 cm height), erect in a more or less branchy way. The leaves are shiny with a beautiful dark green above, paler below opposite, long-stalked, oval, crenelated, embossed. The flowers are white, arranged at the base of the upper leaves. This plant flowering stage (according to this study) is between June and September for both Algerian humid ravines and mountains like Babors, Djurdjura and Mouzaïa. The fruit are surrounded by a persistent calyx and contain shiny dark brown seeds (Quezel & Santa, 1963).

Lemon balm is widespread in the natural flora of Mediterranean region, in North Africa, Southern and Eastern Europe as far as the Caucasus and northern Iran (BAĞDAT & COŞGE, 2006; Dastmalchi et al., 2008; Sadraei et al., 2003; Toth et al., 2003). Lemon balm could be cultivated in Algeria, France, Germany, Italy, Romania, Bulgaria (BAĞDAT & COŞGE, 2006; A Beloued, 2009), southern Slovakia, Moravia (Toth et al., 2003) and North America (BAĞDAT & COŞGE, 2006). The Latin name *Melissa* came from the Greek word meli, melitos which means “members, honeymoon” and probably was named like this since this plant has a strong attraction to bees (Basar & Zaman, 2013).

In traditional medicine, lemon balm could be used in several forms: cream or tea (Herodež et al., 2003) to treat headache, nervousness, gastrointestinal disorders (Boyadzhiev & Dimitrova, 2006; Caniova & Brandsteterova, 2001; Dastmalchi et al., 2008; Fialová et al., 2008; Herodež et al., 2003; Sadraei et al., 2003), bronchitis, depression, hysteria (Dastmalchi et al., 2008; Herodež et al., 2003), rheumatism (Herodež et al., 2003), flatulence, nausea, anemia, vertigo, syncope, asthma, amenorrhea, heart failure, cardiac conduction disturbances, insomnia, epilepsy, psychosis, ulcers, injuries (Dastmalchi et al., 2008).

The studied plant in this work has numerous Algerian vernacular names, like “touroudjan”, “tindjan” and “bararendjabouya”. However, these names could be changed by the changing of regions, i.e. in Berber Algerian region this plant is named “tiferzizwith” (Abdelkader Beloued, 1998; Quezel & Santa, 1963). It is considered to be an important medicinal plant widely used in Algerian traditional medicine. For more details, the leaves are frequently used to prepare a tea, and due to their aromatic, digestive and antispasmodic properties, this plant’s extracts could help to cure both nervous sleep and gastrointestinal disorders (Abdelkader Beloued, 1998). *M. officinalis* has also been reported to contain substances that inhibit protein biosynthesis in cancer cells (Adorjan & Buchbauer, 2010; De Sousa et al., 2004).

In addition, lemon balm (*Melissa officinalis* L.) has various reported curing benefits which may be used as pain sedative, carminative agent, antispasmodic, anti-inflammatory, antiviral and antioxidant agent (De Sousa et al., 2004; Herodež et al., 2003; Lamaison et al., 1991; Lin et al., 2012; Pino et al., 1999; Ribeiro et al., 2001; Tagashira & Ohtake, 1998). Also, it was mentioned that lemon balm essential oil has antibacterial, anti-parasitic, antihistamine and antifungal activity (Hussain et al., 2011; Mencherini et al., 2007; Romeo et al., 2008).

This study is devoted to the phytochemical valuation of lemon balm (*Melissa officinalis* L.) leaves, hence, identifying the main non-volatile chemical constituents (polyphenols including flavonoids) of lemon balm leaves extracts, as well as the evaluation of their biological activities: antimicrobial and antioxidant.

## Materials and Methods

### Plant Material

Samples of *Melissa officinalis* L. plant were randomly collected during May 2018 at Algiers (Algeria). The plant material was identified by the botanical department of the National Institute Agronomic of Algiers (NIA), Algeria.

### Solvent Extraction

The extraction of the polyphenols compounds begins with solvents of increasing polarity which makes it possible to separate the free forms, the esters then the heterosides.

#### Extraction of Flavonic Aglycones and Phenolic Acids

The used technique flavonic aglycones and phenolic acids extraction were extracted with diethyl ether solvent (Lebreton et al., 1967). We introduced 2 g of dry leaves of lemon balm in 160 mL of 2 N hydrochloric acid at 40 °C. The medium is regularly stirred and oxidized by blowing air every 10 min.

After cooling, the solution thus obtained is extracted with 25 mL of diethyl ether 3 times successively in a separating funnel. The dry residues of the 2 ethereal and butanolic phases are recovered in 5 mL of ethanol each and then stored in glass tubes in the refrigerator for analyses.

#### Extraction of Heterosides

In this part, different types of phenolic derivatives particularly heterosides (generally exist in plant tissues) were separated, which consists of a hot hydroalcoholic maceration followed by evaporation to dryness (Remesy et al., 1996). Two grams of vegetable powder was subjected to a maceration in 200 mL of a hydroalcoholic solution following a ratio of 70:30 (vol/vol) at 70 °C for 48 h. The dry residue obtained is taken up in 5 mL of ethanol and stored in the refrigerator for analyses.

### High Performance Liquid Chromatography Analysis

The different extracts were put through to the characterization of phenolic compounds by HPLC–UV/DAD analyses, which were carried out with an Agilent 1100 apparatus equipped with a diode array (DAD) UV detector. The analysis was carried out in reverse phase with column C18 (250 × 4.5 mm, 5 μm). The flow rate was 0.8 mL/min, and the temperature was set to 30 °C, and the injection volume selected was 20 μL. The flow rate was fixed at 0.8 mL/min. The chromatographic conditions consist of solvent A: acetic acid 0.2% and solvent B: methanol (HPLC grade), with the following gradient: 0 min: 95% A + 5% B; 40 min: 30% A + 70% B at the end 60 min 95% A + 5% B. Detection was effected at 200 nm, 230 to 260 nm, 320 nm and 365 to 380 nm. The phenolic acids and flavonoids contained in extracts analyses were recognized by comparing the retention times and the UV spectra obtained by those of the standards used.

Standards of free aglycones, phenolic acids and heterosides were injected under the same conditions as the samples.

#### Analysis of C-Glycosides: (Sample of the Butanolic Phase and the Ethanolic Extract)

C-glycosides and heterosides were separated in gradient elution mode with the same apparatus used for the analysis of free aglycones and phenolic acids, at the wavelength *λ* = 380 nm corresponding to the maximum adsorption of these compounds. The mobile phase consists of the two solvents and according to an elution program as follows:

Solvent A: 0.2% (100%) acetic acid.

Solvent B: Acetonitrile/acetic acid 0.2% (80:20%).

### Antimicrobial Activity of Three Flavonic Extracts of Lemon Balm

The antimicrobial activity of lemon balm extracts was tested by the inhibitory activity against Gram-positive bacteria, Gram-negative bacteria, filamentous fungi and yeasts.

#### Disc Diffusion Method

The antimicrobial activity of three flavon extracts was investigated using paper disc diffusion. Bacterial strains were cultured on Muller–Hinton agar (Institute Pasteur, Algeria), and fungi were cultivated on Sabouraud dextrose agar (Institute Pasteur, Algeria). The samples were solubilized in ethanol then spotted on the paper discs, and sterilized before depositing them on the surface of the culture medium. The diameter of the zones of inhibition around each disc (in millimeters, with the diameter of the paper disc) was taken as a measure of antimicrobial activity.

#### Target Microorganisms

- ***a) Microbial Strains***
  Both leaf’s extracts and standard compounds were individually tested against different microorganisms including 3 Gram positive bacteria (*Bacillus subtilis* ATCC 6633, *Micrococcus luteus*, *Staphylococcus aureus* CIP 7625) and one Gram-negative bacteria (*Klebsiella pneumoniae* CIP 8291), 4 filamentous fungi (*Umbelopsisramanniana*, *Aspergillus carbonarius*, *Aspergillus ochraceus*, *Fusaruimoxysporum* CURZA) and 2 yeasts (*Candida albicans* IPA200, *Candida glabrata*). All microorganisms were graciously supplied from stock cultures of the Microbiology Laboratory of the Department of Biology, Ecole Normale Superieure, Algiers, Algeria. The bacterial strains were cultured on Mueller–Hinton agar for 48 h at 37 °C, while fungi and yeasts were propagated on Sabouraud agar at 37 °C for 48 h to 3 days before use. All microorganisms were regenerated twice before use in the manipulations.

### Evaluation of the Free Radical Scavenging Activity by the DPPH Method

The method of Braca et al. (2002) and Chelalba et al. (2020) was used for the determination of the free radical scavenging activity DPPH (1,1-diphenyl-1-picrylhydrazyl). Briefly, 1.5 mL of different concentrations of the extract prepared in ethanol (between 5 and 1000 μg/mL) was added to equal volume of the freshly prepared ethanol/DPPH solution 0.4 mM. The reaction mixture was stirred with vortex and kept in the dark for 30 min. The absorbance was then read at 517 nm with a spectrophotometer UV–Visible (JASCO-V53) apparatus against a blank. α-Tocopherol (vitamin E) and BHT (hydroxytoluenebutyl) were used as standards. The manipulation was repeated three times.

The inhibition capacity of the samples was expressed in percentage by following the equation:

$$\mathrm{\% \;inhibition \;\;capacity }=1 -\left[\left(\frac{{\mathrm{Abs }}_{\mathrm{sample}}}{{\mathrm{Abs }}_{\mathrm{blank}}}\right)\right] \times 100$$

where:

Abs _sample_ is the absorbance of the sample or after 30 min.

Abs _blank_ corresponds to the absorbance of DPPH^•^ solution without extracts.

Antioxidant activity was expressed as percentage of inhibition in relation to control. According to the equation of IC50 value, the concentration of the extract required to scavenge 50% of free radicals was calculated by linear regression of the calculated inhibition percentages according to different sample concentrations prepared. The above protocol was used to study the anti-free radical activity of the different flavonic extracts and compare with standards flavones such as quercetin, rosmarinic acid luteolin-7-glycosides, luteolin.

### Statistical Analyses

All data measurements in this paper were presented as mean ± standard deviation (SD) and were analyzed by one-way analysis of variance (ANOVA), followed by Tukey’s multiple range tests. *p* < 0.05 was considered as the level of significance.

## Results and Discussion

### The Yields of Flavonic Extracts

We obtained a good 0.23% (w/w) yield of the extraction of flavonic aglycones from the greenish-colored butanolic phase containing the heterosides (C-glycosides and O-glycosides). We obtained a low yield of 0.03% for the ethereal phase of greenish yellow color containing flavones, flavonols and phenolic acids and the same quantity 0.03% obtained for the extraction of heterosides. It is the ethanolic phase of reddish color containing anthocyanins and C-glycosides.

### Identification by High Performance Liquid Chromatography (HPLC/DAD)

**Fig. 1:**
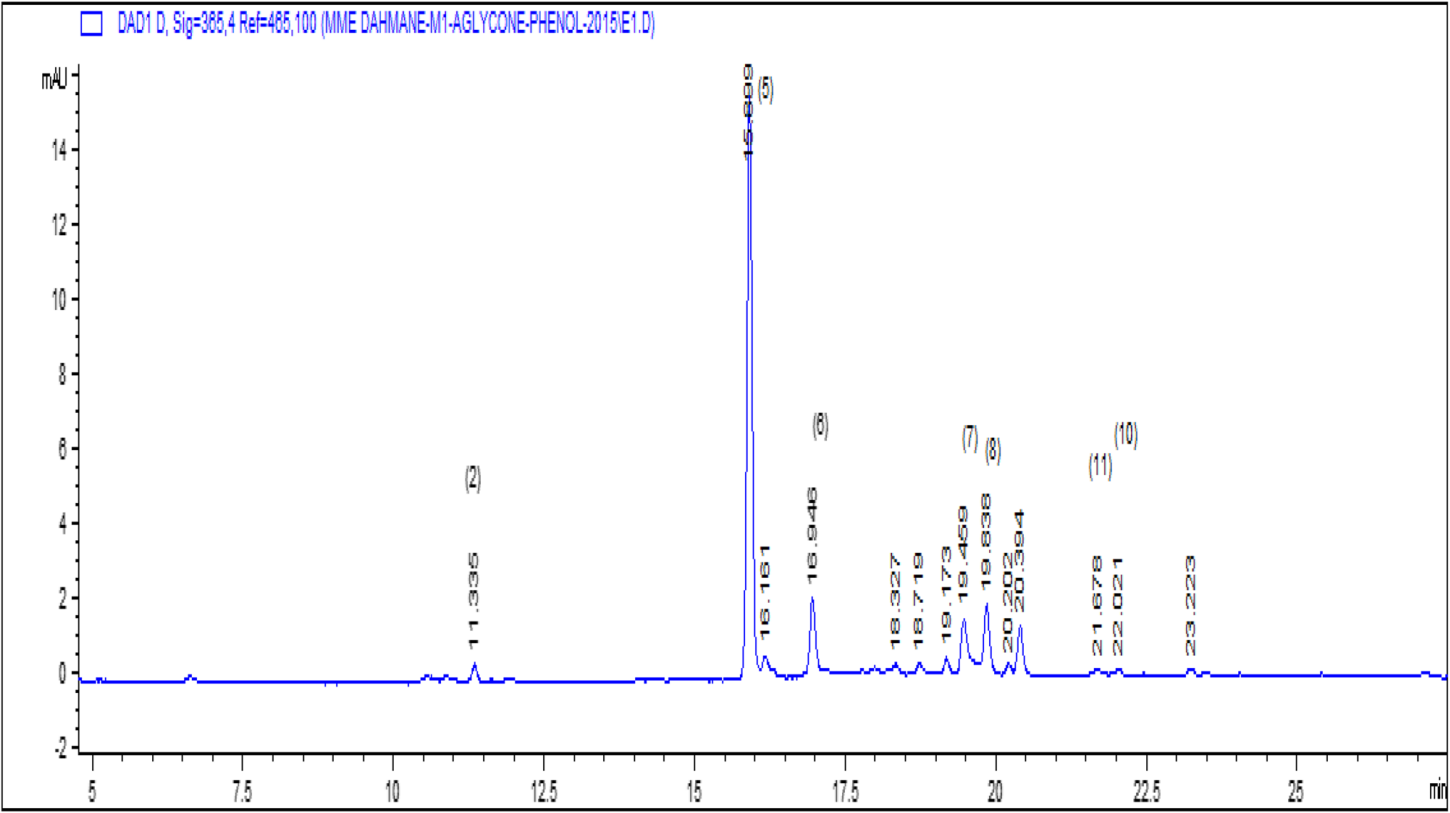
HPLC–DAD chromatogram at 365 nm of the ethereal extract of lemon balm in gradient elution. Peak identification: (2): gallic acid, (5): rosmarinic acid, (6): myricetin, (7): quercetin, (8): luteolin, (11): kaempferol, (10): apigenin

**Fig. 2:**
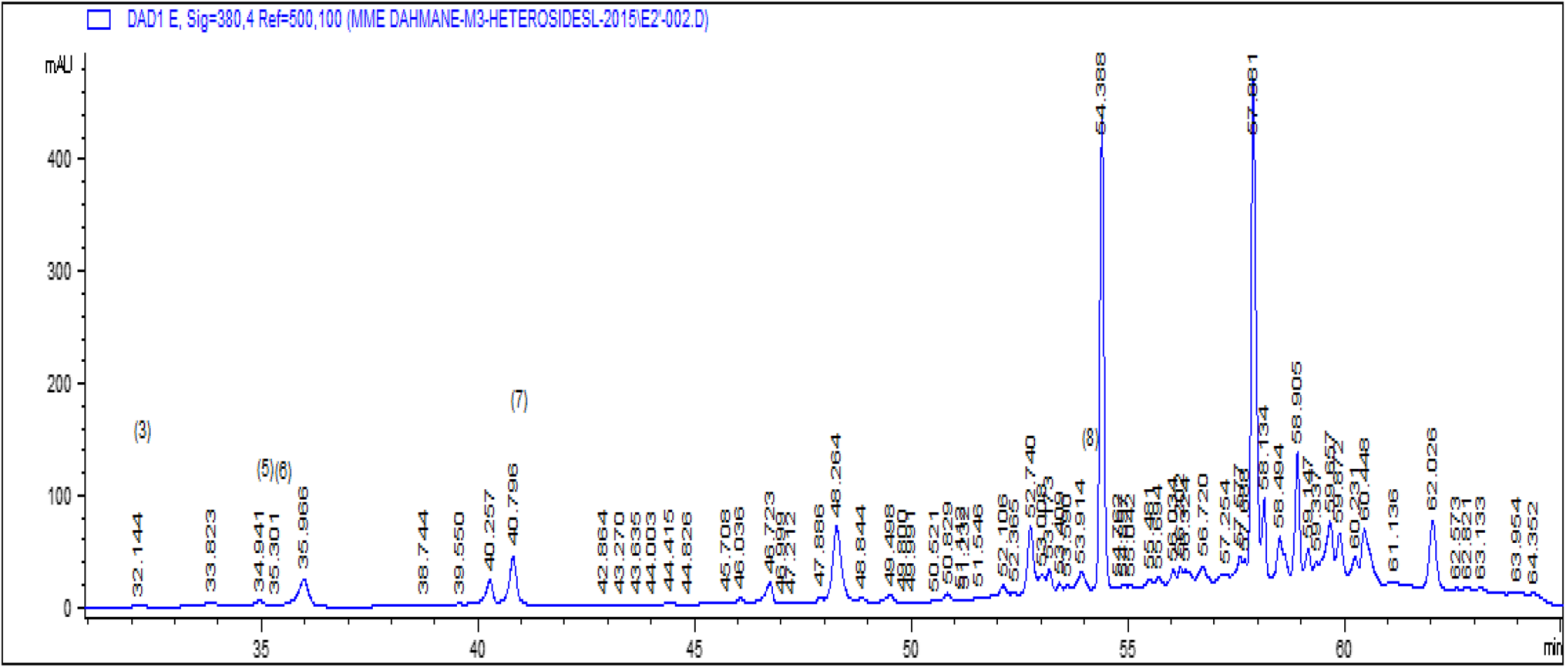
HPLC–DAD chromatogram at 380 nm of the butanol extract of lemon balm in gradient elution mode. Peak identification: (3): vitexin, (5): quercetin-3-β-D-glycoside, (6): luteolin-7-glycoside, (7): apigenin-7-glucoside, (8): isorhamnetrine

**Fig. 3:**
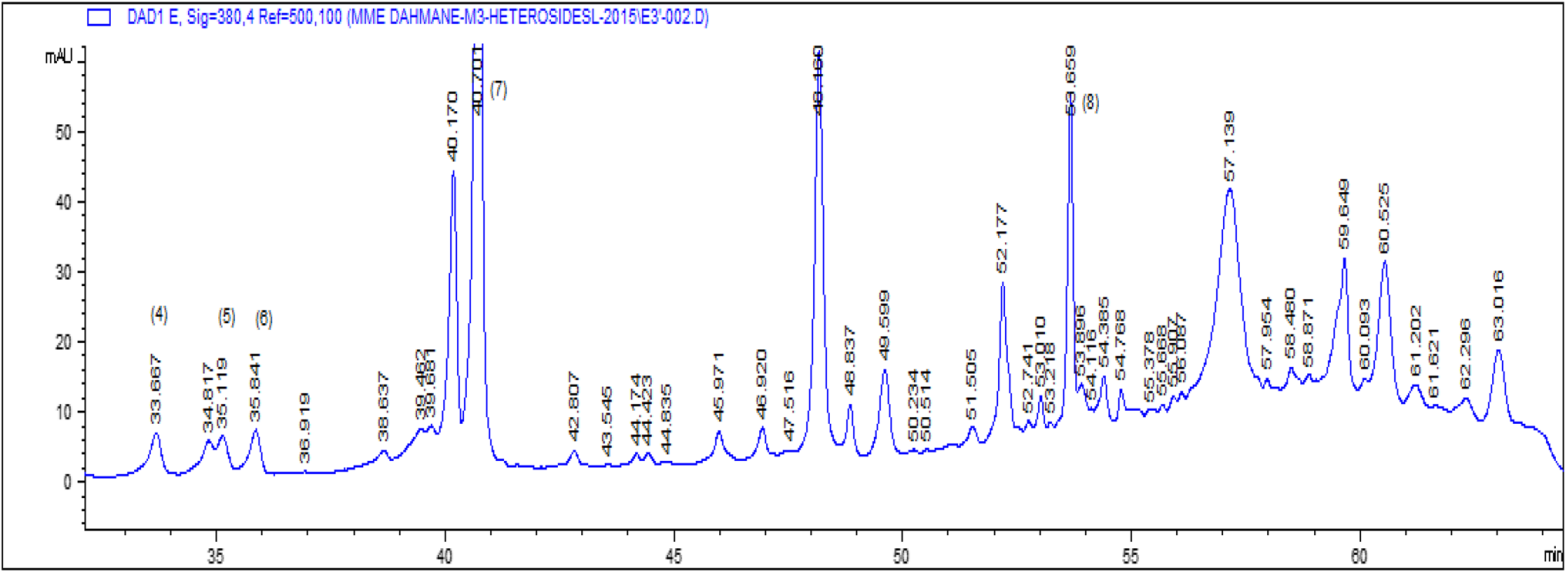
HPLC–DAD chromatogram at 380 nm of the ethanolic extract of lemon balm in gradient elution mode. Peak identification: (4): myricitrin, (5): quercetin-3-β-D-glycoside, (6): luteolin-7-glycoside, (7): apigenin-7-glucoside, (8): isorhamnetrine

- a) Analysis of Free Aglycones and Phenolic Acids (Sample of the Ethereal Phase)
  Flavonic aglycones and phenolic acids were analyzed by gradient elution mode at both wavelengths.
  *λ*1 = 260 nm for phenol acids.
  *λ*2 = 365 nm for flavones and flavonols.
  High performance gradient elution chromatography was used to improve the separation of the studied extract.
  Seven compounds have been identified with a percentage of 82.97% of the overall content of the extract of the ethereal phase, among which we have identified two phenolic acids: caffeic acid with a low percentage of 1.58% and a high percentage rosmarinic acid with 55.39%.
  The main compounds of the aglycone family are present with five compounds such as myricetin 7.77%, quercetin 8.32%, luteolin 7.86%, kaempferol 1.11% and apigenin with 0.94% (Table 1).
  The results of analysis of the extracts have shown the presence 26.01% of the overall content of three flavonols (myricetin, quercetin and kaempferol) and two flavones (luteolin and apigenin). The phenolic acids have an overall relative content of 56.97%. This extract essentially contains rosmarinic acid with a high percentage of 55.39% (Table 1).
  The sedative, carminative, antispasmodic, anti-inflammatory, antiviral and especially antioxidant properties of lemon balm are attributed to rosmarinic acid (Boyadzhiev & Dimitrova, 2006; Caniova & Brandsteterova, 2001; Dastmalchi et al., 2008; Fialová et al., 2008; Ribeiro et al., 2001; Toth et al., 2003; Ziaková & Brandšteterová, 2003). The rosmarinic acid content obtained from our study of lemon balm harvested in Algiers is very high (55.39%) compared to those cited in the bibliography that varies just from 0.5 to 4.75% depending on the studies (Caniova & Brandsteterova, 2001; Lamaison et al., 1990; Toth et al., 2003; Wang et al., 2004).
- b. Identification of Heterosides (C-Glycosides and O-Glycosides): (Sample of the Butanolic Phase and the Ethanolic Phase).
  Analysis of heterosides was used by high performance liquid chromatography in gradient elution mode at the wavelength *λ* = 380 nm corresponding to the maximum adsorption of these compounds, for the identification of heterosides given the structural complexity of these compounds.
  The results obtained are collected in Tables 1 and 2 and illustrated by the chromatogram Figs. 1 and 2. This method of analysis allowed us to identify only 5.25% of the overall content of the butanol phase extract.
  The compounds identified are vitexin (0.04%), quercetin-3-β-D-glucoside (0.29%), luteolin-7-glucoside (1.38%), apigenin-7-glucoside (1.80%) and isorhamnetine (1.74%).
  The extract of the ethanolic phase of cold maceration of lemon balm leaves allowed us to identify 23.85% of the overall content of this extract; on the other hand, the overall content of the extract of the butanolic phase is only low 5.25%.
  The content of the ethanolic phase is higher than that of the butanolic phase.
  The extract of the ethanolic phase of cold maceration of lemon balm leaves allowed us to identify 23.85% of the overall content of this extract. The main compounds identified are myricitrin (1.09%), quercetin-3-β-D-glucoside (0.86%), luteolin-7-glucoside (1.06%), apigenin-7-glucoside (16.07%) and isorhamnetine (4.77%) (Table 2).
  We have identified in the three flavonic extracts of officinal lemon balm 13 main compounds in which:
  2 phenolic acids: caffeic acid and rosmarinic acid;
  5 aglycones: 3 flavonol: myricetin, quercetin and kaempferol;
  2 flavones: luteolin and apigenin;
  6 heterosides: myricitrin, quercetin-3-B-D-glucoside, luteolin-7-glucoside, apigenin-7- glucoside, isorhamnetine and vitexin.
  The families of phenolic acids are present only with two compounds, rosmarinic acid with a high content of 55.39% and low content of caffeic acid 1.58% (Fig. 1).
  The aglycone families are present with five compounds quercetin, luteolin, myricetin, kaempferol and apigenin with levels respectively (8.32, 7.86, 7.78, 1.11 and 0.94%).
  The heterosides family are present by six compounds. The results of the butanolic phase determined the trace amount of compounds vitexin (0.04%) which is absent in the ethanolic extract. On the other hand, the ethanolic extract made it possible to identify the compound myricitrin (1.09%) which is absent in the butanolic extract, quercetin-3-β-D-glucoside, luteolin-7-glucoside, apigenin-7-glucoside. Isorhamnetine is present in both extracts. High content was obtained in the ethanolic extract with (0.86, 1.06, 16.07 and 4.77%) respectively (Figs. 2 and 3). Many authors have reported the predominance of rosmarinic acid in this species (Adinee et al., 2008; Birdane et al., 2007; Hussain et al., 2011; Romeo et al., 2008; Toth et al., 2003; Ziaková & Brandšteterová, 2003). Luteolin derivatives have also been reported in phenolic compounds of *M. officinalis* (Ziaková & Brandšteterová, 2003)*.*
  Phytochemicals are biologically active compounds that are rich in phenols, flavonoids, anthocyanin, minerals (Abdellatif et al., 2021), and antioxidants and can be used for medicinal and health purposes (Noormohammadi & Shamaei, 2022) (Noormohammadi & Shamaei, 2022; Paul et al., 2021). *M. officinalis* plant is resistant to begomovirus infection and Biopharma Sectors with a major components acid rosmarinic.

**Table 1:** The compounds identified by HPLC–DAD of the extract of the ethereal phase of lemon balm in gradient elution analysis mode

*λ* = 365 nm			*λ* = 260 nm		
Flavonic aglycones			Phenolic acids		
*t* (min)	Identified compounds	Relative contents%	*t* (min)	Identified compounds	Relative contents%
16.946	Myricetin	7.78	11.33	Caffeic acid	1.58
19.46	Quercetin	8.32	15.89	Rosmarinic acid	55.39
19.83	Luteolin	7.86	-	-	-
21.67	Kaempferol	1.11	-	-	-
22.02	Apigenin	0.94	-	-	-

**Table 2:** The heterosides identified by HPLC–DAD of the extract of the butanolic and ethanolic phases of lemon balm in gradient elution analysis mode

	Butanolic phase		Ethanolic phase		
*t* (min)	Identified compounds	Relative contents%	*t* (min)	Identified compounds	Relative contents%
32.14	Vitexin	0.04	33.66	Myricitrin	1.09
34.94	Quercetin-3-B-D-glucoside	0.29	35.11	Quercetin-3-β-D-glucoside	0.86
35.30	No identified	0.09	35.84	Luteolin-7-glucoside	1.06
35.96	Luteolin-7-glucoside	1.38	40.70	Apigenin-7-glucoside	16.07
40.79	Apigenin-7-glucoside	1.80	53.65	Isorhamnetin	4.77
53.91	Isorhamnetin	1.74		-

### *Antioxidant Activity *In Vitro* of Flavonic Extracts of Lemon Balm*

The ability of processed of lemon balm extracts to scavenge free radical DPPH^.^ is presented in Table 3. The flavon standards tested are: luteolin-7-glycosides, luteolin, rosmarinic acid and quercitin. The results of flavon extracts showed similar anti-free radical activities as those of the flavon standards.

**Table 3:** Antioxidant activities in vitro of the non-volatile fraction of lemon balm by scavenging DPPH radicals

Fractions	Samples	IC50 (µg/mL)
Non-volatile fraction Flavonic extracts	Aglycones and phenolic acids	2.78 ± 0.02
	C-glycosides and anthocyanins	1.76 ± 0.03
	Heterosides	2.19 ± 0.05
Flavonic standards	Luteolin-7-glycosides	1.29 ± 0.06
	Luteolin	6.81 ± 0.05
	Rosmarinic acid	0.45 ± 0.02
	Quercitin	0.87 ± 0.01

The extract of the C-glycosides and anthocyanins family (1.76 ± 0.03 µg/mL), the extract of the heterosides family (2.19 ± 0.05 µg/mL), the extract of the aglycone family and the acids phenolics (2.78 ± 0.02 µg/mL) showed excellent DPPH free radical scavenging activity.

The antioxidant power of the flavonic extracts increases from the C-glycoside family and the anthocyanins (1.76 µg/mL) and then the IC50 of the extract from the heterosides family (2.19 µg/mL) more than the IC50 of the extract of the family of aglycones and phenolic acids (2.78 µg/mL).

It is extremely important to point out that there is a positive correlation between the potential for antioxidant activity and the amount of phenolic compounds in the extracts.

The antioxidant effect of plants is attributed to the combination of phytochemicals or single component of the plant extracts. The phytochemicals responsible for antioxidant properties mainly are phenolic acids, flavonoids, glycosides, saponins, polysaccharides, stilbenes and tannins (Maheo et al., 2022; Renuka & Jeyanthi, 2021). Herbal plant sources consist of important phyto-constituents such as polyphenols, flavonoids, alkaloids and other components which can against defective cellular metabolism and regulate its functional property.

The active compounds from the plants could be recorded and systematically validated to increase the immune system.

Plant-based infusion has fewer side effects or no side effects. Anti-Covid derived from plant sources have lesser side effects and offered cost effective management of Covid through nutrient supplementation. In future researches, we stading effects the extracts derived *M. officinalis* for Covid-19 (Galanakis et al., 2020).

Rosmarinic acid separated from the *Salvia verticillata* (Shanaida et al., 2021) and demonstrated the promising anti-SARS-CoV-2 and antioxidant properties. Chemical composition of the polyphenols in genus *Salvia* determined by Koshovyi et al. is characterized by dominating rosmarinic acid, caffeic acids and flavonoids such as cosmosiin, cynaroside, hispidulin and cirsimaritin (Galanakis, 2021; Galanakis et al., 2020).

The extract from the *M. officinalis plant* demonstrated the valuable antioxidant activity (IC_50_ = 2.78 μg/mL) against DPPH free radical, and it correlated with the high amount of rosmarinic acid. The high antioxidant activity of rosmarinic acid attracted a great interest as the possible therapeutic agent against free radical mediated disorders (Galanakis, 2021).

The abovementioned effect of this hydroxycinnamic acid is considered due to the relatively easy abstraction of hydrogen atoms of the OH-groups on the rings A and B in the presence of free radicals. Thus, prevailing of rosmarinic acid among the revealed polyphenols of the studied *M. officinalis* plant could be related to its high antioxidant potential. Many researchers point many positive biological activities of polyphenols found in the Lamiaceae representatives and other plants’ sources^.^

### Results of the Antimicrobial Activity of Extracts from the Leaves of Lemon Balm

The results of the study of antibacterial and antifungal activities are reported in Table 4. According to Cabezas-Cruz et al. (2017), the inhibition zone > 14 mm indicates very active extracts.

**Table 4:** Measurement of the antibacterial activity of three flavonic extracts from lemon balm leaves by the paper disc method

		Diameter of inhibition of extracts (mm)			
Bacteria tests		Ethereal phase	Phase butanolic	Ethanolic macerate phase	Gentamicin Positive control
Grampositif bacteria	*Micrococcus luteus* *Bacillus subtilis* *Staphylococcus aureus*	14 14 18	15 14 18	41 25 35	- - 25
Gram negative bacteria	*Klebsiella pneumoniae*	-	-	28	24
Filamentous fungi	*Umbelopsisramanniana* *Aspergillus carbonarius* *Aspergillus ochraceus* *Fusaruimoxysporumabedinis*	- 36 - -	- 34 - 16	9 - 20 38	- 17 - -
Yeasts	*Candida albicans* *Candida glabrata*	- -	- -	8 9	20 -

The values of the diameters of the zones of inhibition include that of the paper disc which is 6 mm

-: absence of inhibition zone detected

^*^The reference is antifungal amphotericin B for filamentous fungi and yeast to Itraconazole

We noticed that the three samples of flavonic extracts from lemon balm leaves are active against Gram-positive bacteria (*Micrococcus luteus* (Ml), *Bacillus subtilis* (Bs) and *Staphylococcus aureus* (Sa)) with areas inhibition varying from 14 to 41 mm in diameter and even greater than that of the control antibiotic (Gentamicin). Depending on the performance scale, we can therefore consider these strains to be very sensitive. They are less active and sometimes inactive against Gram-negative bacteria (*Klebsiella pneumoniae* (Kp)).

We deduce that the flavonoids and phenolic acids extracted from lemon balm leaves have good antibacterial activity against these strains.

The ethanolic macerate has very good activity (35 mm) against the bacterium *Staphylococcus aureus* (Sa), even greater than that of the reference antibiotic, Gentamicin (25 mm).

The bacteria *Staphylococcus aureus* is responsible for skin and subcutaneous infections and foodborne illnesses. The richness of this plant in flavonoids and phenolic acids known by their antispasmodic effects, their antibacterial, antifungal and antiviral properties justifies its use in traditional medicine in the treatment of certain skin diseases and against spasmodic colitis.

The ethereal extract and the butanol extract show similar activity against 3 Gram-positive bacteria (*Micrococcus luteus* (Ml), *Bacillus subtilis* (Bs) and *Staphylococcus aureus* (Sa)). The diameters of the zones of inhibition are practically equal and slightly smaller than that of the control antibiotic (Gentamicin). In contrast, no activity was detected against the Gram-negative bacterium *Klebsiella pneumoniae* (Kp).

The results of the antifungal tests of the three flavonic extracts contained in the leaves of *Melissa officinalis* with respect to fungi and yeasts are collated in Table 4.

We note that the polyphenols extracted from the leaves of lemon balm have a very low activity against two yeasts *Candida albicans* (Ca) and *Candida glabrata* (Cg). On the other hand, the ethereal extract and the butanolic extract do not exhibit any activity against the fungi *Umbelopsisramanniana* (Ur) and *Aspergillus ochraceus* (Ao) as well as against the two yeasts *Candida albicans* (Ca) and *Candida glabrata* (Cg).

The ethereal extract and the butanolic extract exhibit very good activity against the filamentous fungus *Aspergillus carbonarius* (Ac) with zones of inhibition of 36 and 34 mm respectively; they exhibit greater activity than that of amphotericin B, the reference antifungal (17 mm).

## Conclusions

This study focuses on quantitative and qualitative analyses of *Melissa officinalis*, growing in Northern Algeria, for the first time, in addition to giving an overview of some biological efficacy. We note that the polyphenols extracted from the leaves of lemon balm have very good activity towards Gram-positive bacteria and less activity towards Gram-negative bacteria. Filamentous fungi and very low activity vis-à-vis two yeasts tested. The results of the current work may be beneficial and can be used as a database for researchers and specialists and to enrich the aromatic and medicinal herbs database.

According to revealed chromatographic profiles, the herb of *M. officinalis* can be considered mainly as a valuable source of rosmarinic acid which possesses the proven therapeutic properties to become a potential drug for preventing and treating many diseases caused by oxidative stress, antimicrobial effect or infection. The common presence of such valuable compounds as hydroxycinnamic acids (rosmarinic and caffeic) and aromatic monoterpenoids (citral, citronellal and p-cymene) can be considered as an important chemotaxonomic feature of the *M. officinalis* herb.

*Melissa officinalis* l. is widely used for therapeutic and non-therapeutic purposes that trigger its significant value. Various combinations and numerous medicinal properties of its extract, oil and leaves demand further and more studies about the other useful and unknown properties of this multipurpose plant.

## Data Availability

Data will be made available on reasonable request.
